# Effect of meloxicam and lidocaine administered alone or in combination on indicators of pain and distress during and after knife castration in weaned beef calves

**DOI:** 10.1371/journal.pone.0207289

**Published:** 2018-11-30

**Authors:** Daniela M. Meléndez, Sonia Marti, Edmond A. Pajor, Pritam K. Sidhu, Désirée Gellatly, Diego Moya, Eugene D. Janzen, Johann F. Coetzee, Karen S. Schwartzkopf-Genswein

**Affiliations:** 1 Department of Production Animal Health, University of Calgary, Calgary, Alberta, Canada; 2 Lethbridge Research Centre, Agriculture and Agri-Food Canada, Lethbridge, Alberta, Canada; 3 Department of Ruminant Production, IRTA, Caldes de Montbui, Barcelona, Spain; 4 Department of Anatomy and Physiology, Kansas State University, Manhattan, KS, United States of America; 5 Department of Large Animal Clinical Sciences, University of Saskatchewan, Saskatoon, Saskatchewan, Canada; University of Bari, ITALY

## Abstract

To assess the effect of meloxicam and lidocaine on indicators of pain associated with castration, forty-eight Angus crossbred beef calves (304 ± 40.5 kg of BW, 7–8 months of age) were used in a 28 day experiment. The experiment consisted of a 2 × 2 factorial design where main factors included provision of analgesia and local anaesthesia. Analgesia consisted of: no-meloxicam (**N**; *n* = 24) single s.c. administration of lactated ringer’s solution and meloxicam (**M**; *n* = 24) single dose of 0.5 mg/kg of s.c. meloxicam. Local anesthesia consisted of: no-lidocaine (**R**; n = 24) ring block administration of lactated ringer’s solution or lidociane (**L**; *n* = 24) ring block administration of lidocaine. To yield the following treatments: no meloxicam + no lidocaine (**N-R**; *n* = 12), no meloxicam + lidocaine (**N-L**; *n* = 12), meloxicam + no lidocaine (**M-R**; *n* = 12) and meloxicam + lidocaine (**M-L**; *n* = 12). Salivary cortisol concentrations were lower (lidocaine × time effect; *P* < 0.01) in L calves than R calves 0.5 and 1 hours after castration, while concentrations were lower (meloxicam × time effect; *P* = 0.02) in M calves than N calves at 2, 4 and 48 hours. The serum amyloid-A concentrations were greater (lidocaine × time effect; *P* < 0.01) in R calves than L calves on days 1, 3, 21 and 28 after castration. Haptoglobin concentrations were greater (meloxicam × time effect; *P* = 0.01) in N calves than M calves 24 and 48 hours after castration. Lower (lidocaine effect; *P* < 0.01) visual analog scale (VAS) scores, leg movement frequencies and head movement distance were observed in L calves than R calves at the time of castration. Escape behaviour during castration was lower (lidocaine effect; *P* < 0.05) in L calves than R calves based on data captured with accelerometer and head gate devices. Scrotal circumference had a triple interaction (lidocaine × meloxicam × time; *P* = 0.03), where M-R calves had greater scrotal circumference than M-L calves 28 d after castration, but no differences were observed between both groups and N-R and N-L calves. No differences (*P* > 0.05) were observed for average daily gain (ADG), weights or feeding behaviour. Overall, both lidocaine and meloxicam reduced physiological and behavioural indicators of pain. Although there was only one meloxicam × lidocaine interaction, lidocaine and meloxicam reduced physiological and behavioural parameters at different time points, which could be more effective at mitigating pain than either drug on its own.

## Introduction

The public is increasingly concerned about the conditions in which livestock are raised [1], and especially about routine painful husbandry procedures such as castration, dehorning and branding [2]. For instance, although castration has been previously reported to cause physiological and behavioural changes indicative of pain and distress [3], this procedure is commonly done without the use of pain control [4].

Canada has codes of practice specific to farmed animals which identify recommended best management practices. The Canadian Beef Codes of Practice [5] recommend using pain control under veterinary advisement when performing painful procedures. As of January 2018, it is a requirement of the Canadian Beef Codes of Practice to castrate calves 6 months of age or older with the use of pain mitigation. However, pain mitigation is a vague term as it can refer to the use of only an anaesthetic, only an analgesic or the combination of both. Currently, there are no standard pain mitigation protocols as it is challenging to identify a protocol which is practical, cost effective and animal welfare friendly. In addition, there is a lack of drugs labelled for pain control associated with castration, with the exception of oral meloxicam.

Drugs commonly used to mitigate pain at the time of castration include local anaesthetics, to block the conduction of pain signaling during a painful procedure, and analgesics, to mitigate the pain associated with inflammation as a result of tissue damage. Lidocaine is a local anaesthetic that is frequently used in veterinary medicine due to its fast onset of action (5 to 10 minutes) and low toxicity, which works by blocking sodium channels in the neurons that carry nociceptive information [6]. Meloxicam is a non-steroidal anti-inflammatory drug (NSAID) approved for use in cattle in Canada, but not in the United States. Meloxicam is an attractive option for use in production animals due to its ease of administration (s.c. or oral) and long lasting half-life (s.c.: 22 ± 3 hours; oral: 27 hours) [7]. Meloxicam inhibits COX-2 enzymes, which convert arachidonic acid into prostaglandins, which are pro-inflammatory substances [8]. Although previous studies have assessed the pharmacokinetics of intravenous and oral meloxicam [9], to our knowledge there are no studies assessing the pharmacokinetics of s.c. meloxicam in beef cattle.

Different physiological, behavioural and neuroendocrine parameters have been previously used to assess pain, stress and inflammation associated with castration. Cortisol has been reported to elicit a greater cortisol response compared to uncastrated calves [10–12], while production parameters have been reported to decrease as the age of castration increases [13]. In addition, acute phase proteins have been evaluated after castration as indicators of infection, inflammation or trauma [14], with haptoglobin levels peaking 2 to 3 days castration [10,15]. Neuropeptides and catecholamines have also been assessed after castration with substance P [16] and epinephrine/norepinephrine [17] reaching peak concentrations 45 minutes and 2 minutes after castration, respectively.

Additional parameters used to assess pain associated with castration include: infrared thermography of the eye, [17], heart rate [17,18], heart rate variability [19], electrodermal activity [20], electroencephalography [21], visual analog scale (VAS) [22], behaviour during castration [23,24], accelerometers [25], pain related behaviour [26,27], stride length [28,29], and the von Frey anesthesiometer [30].

In this study, physiological and behavioural parameters were collected to objectively assess pain and inflammation associated with castration. The objective of this study was to evaluate the effect of meloxicam, lidocaine, and the combination of meloxicam and lidocaine on indicators of pain, and to describe the pharmacokinetics of s.c. meloxicam in 7–8 month old beef calves. We hypothesise that the combination of drugs would be more effective at reducing markers of pain associated with knife castration than either drug administered alone, and that the pharmacokinetics of meloxicam would be similar when administered alone or in combination with lidocaine.

## Materials and methods

This protocol was approved by the Animal Care Committees of Lethbridge Research and Development Centre (ACC number 1522) and the University of Calgary (AC15- 0138). Animals were cared for in accordance with the Canadian Council of Animal Care [31].

### Animal housing and management

Forty-eight Angus crossbred beef calves (304 ± 40.5 kg of body weight (BW) and 7 to 8 months of age) were used in a 28 day (d) experiment. Calves were divided into two groups of 24 animals and each group was castrated on different days, 7 d apart. Upon weaning, calves were vaccinated with a 7-way clostridial vaccine (Ultrabac/Somubac, Zoetis Canada Inc., Kirkland, Canada), and housed in 4 experimental pens (12 calves/pen) for a 3 week adaptation period prior to the start of the trial. Pens (40.2 m × 27.4 m) contained straw bedding, ad libitum water provided through a centrally located water system and ad libitum feed consisting of a total mixed ration of 80% barley silage, 17% dry-rolled barley and 3% supplement with vitamins and minerals to meet beef cattle nutrition requirements [32].

Calves were equally distributed by weight into pens and randomly assigned to treatments. The day of castration, calves were restrained in a hydraulic squeeze chute (Cattlelac Cattle, Reg Cox Feedmixers Ltd, Lethbridge, Alberta, Canada) where blood, saliva and hair samples were collected and castration took place. The experiment consisted of a 2 × 2 factorial design where main factors included analgesia and local anesthesia. Analgesia consisted of: non-medicated (**N**; *n* = 24) single s.c. administration of lactated ringer’s solution (Lactated Ringer’s Irrigation, Baxter Canada, Mississauga, Ontario, Canada) and medicated (**M**; *n* = 24) single dose of 0.5 mg/kg of s.c. meloxicam (Metacam 20 mg/mL, Boehringer Ingelhein, Burlington, Ontario, Canada). Local anaesthesia consisted of: non-medicated (**R**; n = 24) ring block administration of lactated ringer’s solution (Lactated Ringer’s Irrigation, Baxter Canada, Mississauga, Ontario, Canada) or medicated (**L**; n = 24) ring block administration of lidocaine (lidocaine hydrochloride 20 mg/mL and epinephrine 0.01 mg/mL, Bimeda, Ontario, Canada), to yield the following treatments: no meloxicam + no lidocaine (**N-R**; *n* = 12), no meloxicam + lidocaine (**N-L**; *n* = 12), meloxicam + no lidocaine (**M-R**; *n* = 12) and meloxicam + lidocaine (**M-L**; *n* = 12). The lidocaine with epinephrine block and the sham block were administered 30 min prior to castration to allow time for lidocaine onset. The ring block consisted of administering 5 ml into each spermatic cord and 20 ml subcutaneously around the neck of the scrotum. Meloxicam and the sham injection were administered s.c. on the neck of the calves immediately after the lidocaine block 30 min before castration. The same veterinarian performed the scrotal lidocaine block, and the surgical castration on all the calves by making a latero-lateral incision on the scrotum with a Newberry castration knife (Syrvet Inc., Waukee, IA) and an emasculator was used to crush and cut the spermatic cords.

### Measurements of biomarkers of pain, stress and inflammation and sample collection

Sampling time points included 24 hours (h) before castration (d -1), immediately before castration (T0), and 0.5, 1, 2, 4, 24, 48, 72, 144, 336, 505, 672 h after castration which is equivalent to T0, 30, 60, 120 and 240 min and d 1, 2, 3, 6, 14, 21 and 28 after castration.

#### Saliva and hair cortisol

Salivary samples were collected on d -1, T0, 0.5, 1, 2, 4, 24, 48, 72, 144, 336, 505, 672 h after castration. Salivary samples were collected using a cotton swab that was stored in a plastic tube and frozen at– 20° C for further analysis [33]. Salivary cortisol concentrations were quantified using an enzyme immunoassay kit (Salimetrics, State College, PA). The inter-assay coefficient of variation (CV) was 10.3% while the intra-assay CV was 9.2%. Hair from the forehead of the calves was clipped on d– 1 and 672 h and stored in plastic bags at room temperature and samples were handled as described by Moya et al. [34]. Cortisol was quantified using an enzyme-immunosorbent assay (Salimetrics, State College, PA). The intra-assay and the inter-assay’s CV were 9.7% and 12.1% respectively.

#### Serum amyloid-A (SAA), haptoglobin and white blood cell count

Blood samples were collected from all calves through jugular venipuncture on d -1, T0, 0.5, 1, 2, 4, 24, 48, 72, 144, 336, 505, 672 h after castration.

Blood samples for serum amyloid-A (SAA) and haptoglobin were collected into 10-ml non-additive tubes (BD vacutainer; Becton Dickinson Co., Franklin Lakes, NJ), centrifuged for 15 min at 1.5 × *g* at 4°C and the serum was decanted and frozen at -80 ºC for further analysis [35]. The inter-assay CV for haptoglobin was 10.8%, while SAA intra-assay and inter-assay CV were 8.8% and 10.3%, respectively.

Blood samples for white blood cell count were collected into 6-ml EDTA tubes (BD vacutainer; Becton Dickinson Co., Franklin Lakes, NJ) and were measured using a HemaTrueHematology Analyzer (Heska, Lobeland, Co).

#### Scrotal area temperature (SCT)

Images of the scrotum and its surrounding area were collected on d -1, T0, 0.5, 1, 2, 4, 24, 48, 72, 144, 336, 505, 672 h after castration. A FLIR i60 infrared camera (FLIR Systems Ltd., Burlington, ON, Canada) was used to capture infrared images of the scrotal area at a distance of 1 m from the scrotal area, and FLIR Tools version 5.1 (FLIR Systems Ltd.) was used to delineate the scrotal area and to record the maximum temperature [36]. An emissivity coefficient of 0.98 was used to analyze the images.

#### Scrotal circumference

The area of the scrotum was evaluated 72, 144, 336, 505 and 672 h after castration using a scrotal tape (Reliabull, Lane Manufacturing, Denver, CO) to measure scrotal circumference applied on the widest part of the scrotum [37].

#### Rectal temperature (Temp)

A digital thermometer (M750 Livestock Thermometer, GLA Agricultural Electronics, San Luis Obispo, CA) was used to collect rectal temperature on d -1, T0, 0.5, 1, 2, 4, 24, 48, 72, 144, 336, 505, 672 h after castration.

#### Weight

Calves were weighed in a hydraulic squeeze chute (Cattlelac Cattle, Reg Cox Feedmixers Ltd, Lethbridge, Alberta, Canada) to obtain the initial (d -1) and final (d 28) BW. The average daily gain (ADG; kg/d) was calculated by subtracting the weights obtained on d 28 from the weight obtained on d -1, and dividing the result by 30 which was the number of days in the experiment.

#### Visual analog scale (VAS)

Visual analog scale was collected by two experienced observers (blind to the treatments) which placed a mark along a 10 cm line (far left indicating no pain and far right extreme pain) as an indicator of their perception of the amount of pain calves were experiencing during castration [36].

#### Head movement

Head movement was collected with a video camera placed in front of the head gate during castration to record head movement. An observer blind to treatment used the middle of the hairline of the muzzle as a reference point to track the total head movement distance (cm) during castration using Kinovea (General Public License) version 2 [35].

#### Leg movement and vocalizations

The same observers assessing the VAS scored behaviour such as frequency of urination, defecation, any leg movement and vocalizations at the time of castration [24].

#### Accelerometers and head gate

Escape response was assessed during castration. Briefly, the right and left head gate were equipped with strain gauges to measure the force cattle exerted on the head gate by pushing or pulling, while the chute was equipped with three 1-axis accelerometers (CXL-GP Series, Aceinna, Andover, MA) accelerometers measuring lateral, vertical and horizontal movement. The analog signals (V) from the accelerometer and strain gauges were sent to a computer at a rate of 100 samples/s. Data from the accelerometers was added by animal to obtain an overall acceleration force, and the data from the left and right head gate were added by animal to obtain an overall head gate force [35]. Data from d -1 was used as a baseline for each calf, this data was collected after the animal entered the chute and prior to sampling for a 20 second period. Variables included head gate and accelerometer number of peaks between 1 and 2 SD, 2 and 3 SD, and above or below 3 SD above and below the mean, and total area between the mean ± 1 SD, mean ± 2 SD, and mean ± 3 SD. These variables were divided by the time required to castrate each calf.

#### Feeding behaviour

Calves were fitted with a radio frequency ear tags and each pen was equipped with a GrowSafe feed bunk monitoring system (GrowSafe Systems, Airdrie, Alberta, Canada) with 5 feeding tubs which recorded feeding behaviour for each individual calf 24 h a day over a 28 d period (672 h) [26]. The following variables were calculated from the feeding behaviour data recorded per day but were then summarized per week: feeding duration (min/d), dry matter intake (kg/day), feeding rate (g/min), meal frequency (number/d), meal duration (min/meal) and meal size (kg/meal) [15]. As in the previous study, a meal criterion of 300 s was selected as it has been previously used in cattle [38,39].

#### Stride length

Calves were recorded when walking through a 1 x 3 m alley on d-1, immediately after castration, 0.5, 1, 2, 4, 2, 48, 72, 144, 336, 505, 672 h after castration. Stride length was collected as described by Currah et al. [40] however image analysis software differed between studies and in the present study a grid background was not used. Pictures of the back legs were taken by observers blind to the treatments with GOM player (GOM Lab, Gretech Corporation, Seoul, South Korea), and measured using Image J (National Institutes of Health Image, Bethesda, MD).

#### Standing and lying behaviour

Standing and lying behaviour were measured daily using accelerometers (Hobo pendant G, Onset Computer Corporation, Bourne, MA) to determine daily standing and lying percentage, and daily average standing and lying bout durations [41]. Accelerometers were wrapped in plastic to protect them from moisture and in foam to avoid discomfort when placed on the calves using Vet Wrap (Professional Preference, Calgary, Canada) [24]. Accelerometers were placed on the calves on d -1 and changed weekly. Information from days when accelerometers were changed (d 6, 14, 21 and 28) were excluded from the analysis due to incomplete data collection.

#### Pen behaviour

A subset of calves (6 animals/ treatment) was recorded for behavioural assessment. Two experienced observers blind to treatments scored behaviour for a 2 hour period between 5 to 7 h relative to castration on d 0, and at the same time of the day on d 1 and 2 after castration. Focal animal sampling from continuous recordings [42] were used to score frequency of tail flicks, foot stamping, head turning and lesion licking and duration of standing, lying, walking and eating. Behaviours were modified from the ethogram described by Molony et al. [26]. Behaviours were defined as: a) eating: ingesting hay or straw from the ground or the feeder, b) lying: either lateral (laying with hip and shoulder on the ground with at least 3 limbs extended) or ventral (laying in sternal recumbency with legs folded under the body or one hind or front leg extended) lying, c) walking: walking forward more than 2 steps, d) standing: standing on all four legs, e) foot stamping: hind legs are lifted and forcefully placed on the ground or kicked outwards while standing, f) head turning: head is turned and touches the side of the calf’s body when standing, including head turning to groom, g) tail flicking: forceful tail movement beyond the widest part of the rump when standing, movement to one side is counted as one action, h) lesion licking: head turning to lick the lesion caused by castration while standing [24]. Inter-rater and intra-rater reliability were 0.93 and 0.95 respectively.

#### Meloxicam pharmacokinetics

Meloxicam samples were collected at T0, 1, 4, 24, 48 and 72 h after castration to determine plasma concentrations of meloxicam. Samples were collected through jugular venipuncture into 10-ml lithium heparin tubes (BD vacutainer; Becton Dickinson Co., Franklin Lakes, NJ), centrifuged for 15 min at 1.5 × *g* at 0°C and the serum was stored at -80°C [35]. A subset of 8 samples per treatment were analyzed using high-pressure liquid chromatography (Agilent 1100 Pump, Column Compartment, and Autosampler, Santa Clara, CA, USA) with mass spectrometry detection (LTQ, Thermo Scientific, San Jose, CA, USA) at Iowa State University, College of Veterinary Medicine (Ames, IA).

The plasma concentration vs. time data of meloxicam following s.c. administration of meloxicam and meloxicam + lidocaine were analyzed to determine pharmacokinetic (PK) parameters of meloxicam. The analyses were performed using the software (Phoenix Win-Nonlin 7.0, Certara, Inc. Princeton, NJ, USA). Non-compartment PK approach was applied to the data using a pre-structured model (Model: Plasma 200–202 with uniform weighting) in the software. The slope of terminal phase (λ_z_) of the log plasma concentration vs. time curve was estimated by means of linear regression; while the half-life of the terminal phase (λz_-HL_) was calculated using the following equation: λz_-HL_ = $\frac{0.693}{\lambda_{z}}$

Area under the plasma concentration vs. time curve (AUC) and area under the first moment of the plasma concentration vs. time curve (AUMC) were calculated by use of the log- linear trapezoidal method [18]. Time range from the first measurement (0h) to the last measurement (72h) of drug concentration was used for the calculation of AUC_0-last_ and AUMC_0-last_. The AUC and AUMC were extrapolated to infinity to determine AUC_0-∞_ and AUMC_0-∞_ to account for the total meloxicam exposure to calves [43]. Apparent volume of distribution during terminal phase (V_z-F_) and total systemic clearance scaled by bioavailability (CL_-F_) and mean residence time (MRT) of drug were also determined. Peak plasma concentration (C_max_) and time to achieve peak concentration (T_max_) were determined directly from the data.

### Statistical analysis

A power analysis was done using salivary cortisol and tail flick means, an α of 0.05, a power of 0.08 and the SD observed in a previous study under similar experimental conditions [24]. The power analysis indicated that 6–12 calves per treatment were necessary to detect differences among treatments. Animals were the experimental unit, treatments were mixed within pen and all animals in one pen were castrated on the same day. Calves were divided in two groups and castrated 1 week apart which was added as a covariate in the model. Data was tested for normality with PROC UNIVARIATE (SAS, version 9.4, SAS Inst. Inc., Cary, NC) and physiological data that did not follow a normal distribution was log transformed, while behavioural data was square root + 1 transformed, and percentage data was arcsin transformed. Data was analyzed using the MIXED procedure in SAS (SAS, version 9.4, SAS Inst. Inc., Cary, NC) with meloxicam, lidocaine, time and their interactions as fixed effects and pen and calf within pen as random effects. All data, with the exception of behaviour during castration (VAS, frequency of leg movement, vocalizations, and ERM) hair cortisol and weights, was analyzed using a mixed model for repeated measures, as samples were collected at different time points. The data collected on d-1 was used as a covariate for all physiological parameters and stride length, while data collected the week before castration was used as the baseline for feeding behaviour. Escape response measurements collected on d-1 were used as a baseline for each calf. Urination and defecation were not analyzed as these behaviours were not present during castration. Covariance structures included unstructured, compound symmetry and autoregressive order one. The structure with the lowest Schwarz’s Bayesian criterion was selected as the analysis of choice. The PDIFF option in SAS was used as the post-hoc test to separate the Least Square means. Effect of lidocaine, meloxicam and time were statistically significant when *P* ≤ 0.05.

## Results and discussion

### Markers of inflammation

#### Salivary and hair cortisol

**Fig 1:**
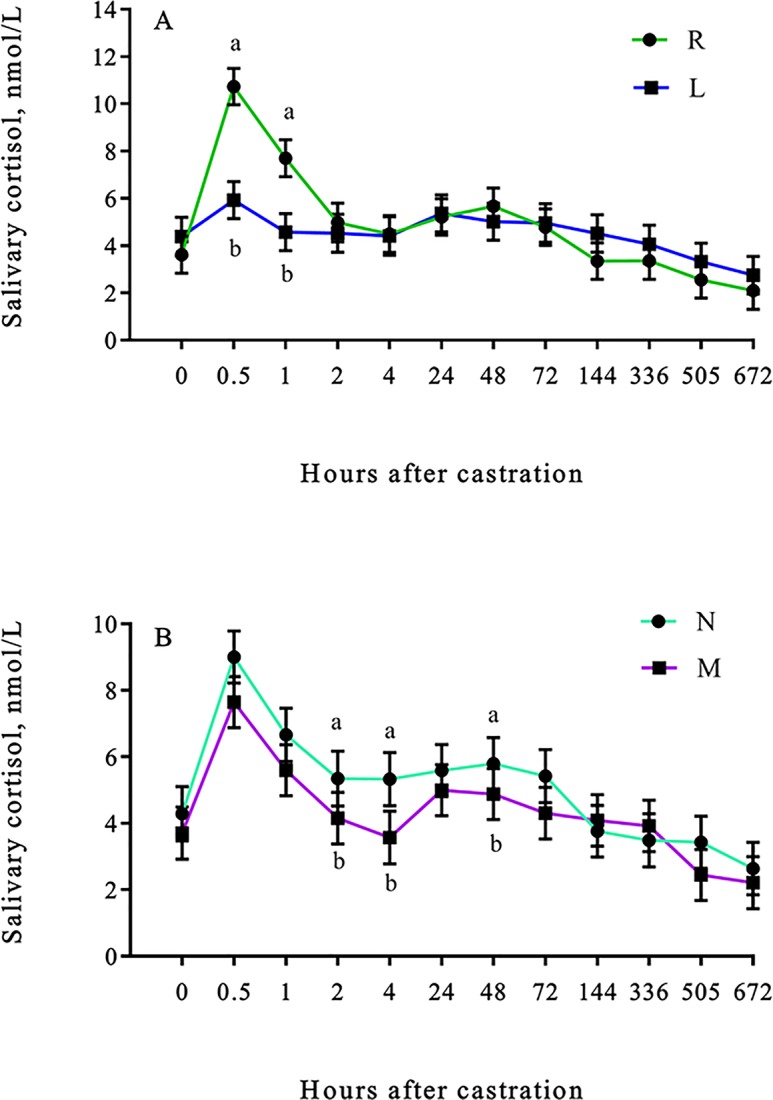
Least square means (±SEM) for salivary cortisol of weaned Angus crossbred calves. Salivary cortisol (nmol/L) of weaned Angus crossbred calves (A) with (L) or without (R) a lidocaine ring block and (B) with (M) or without (N) a single s.c. meloxicam injection. ^a-b^Least square means with differing superscripts differ (*P* ≤ 0.05).

Salivary cortisol concentrations were lower (lidocaine × time effect; *P* < 0.01) in L calves than R calves 0.5 and 1 h after castration (Fig 1A). Salivary cortisol concentrations were also lower (meloxicam × time effect; *P* = 0.02) in M calves than N calves 2, 4 and 48 h after castration (Fig 1B). No differences (*P* > 0.05) were observed for hair cortisol concentrations (Table 1; data in S1 File).

**Table 1:** Least square means (± SEM) of hair cortisol, scrotal temperature (SCT), rectal temperature (Temp), white blood cell count (WBC) and weight (initial BW, final BW and ADG) of weaned Angus crossbred calves with (M) or without (N) a single s.c. meloxicam injection and with (L) or without (R) a lidocaine ring block^1^.

	Treatment^2^							
	R		L			*P-Value*		
Item	N	M	N	M	*SEM*^*3*^	MEL	LID	MEL × LID
Hair cortisol, nmol/L	3.5	3.3	3.1	2.9	0.14	0.72	0.09	0.89
SAA, μg/mL	153	138	147	122	0.09	0.94	<0.01	0.52
Haptoglobin, g/L	1.5	1.6	1.4	1.1	0.09	0.17	0.16	0.12
SCT,°C	34.6	34.7	35.0	34.4	0.03	0.96	0.25	0.21
Temp,°C	39.8	39.7	39.8	39.8	0.07	0.27	0.46	0.28
WBC, × 10^9^/L	10.8^a^	10.8^a^	11.3^a^	9.9^b^	0.27	0.40	0.01	0.02
Scrotal circumference, cm	24.5	24.7	24.1	21.9	1.59	0.19	0.38	0.31
Weight								
Initial BW (d-1), kg	300.1	301.7	303.1	300.6	12.27	0.94	0.97	0.87
Final BW (d 28), kg	320.7	320.6	322.2	318.2	12.65	0.98	0.87	0.88
ADG, kg/d	0.71	0.63	0.64	0.58	0.12	0.51	0.46	0.85

^a-b^Least square means within a row with differing superscripts differ (*P* ≤ 0.05)

^1^Values in the table represent the mean of d 28 for hair cortisol samples; the means of (T0), 0.5, 1, 2, 4, 24, 48, 72, 144, 336, 505, 672 h after castration for scrotal temperature (SCT), rectal temperature (Temp) and white blood cell count (WBC).

^2^ Provision of anaesthesia administered 30 min prior to castration: R: no lidocaine; L: lidocaine; and provision of analgesia administered s.c. 30 min prior to castration N: no meloxicam; M: meloxicam.

^3^The values correspond to non-transformed means, however, the SEM and the *P-*values correspond to ANOVA analysis using log transformed data for hair cortisol and SCT.

#### Acute phase proteins

**Fig 2:**
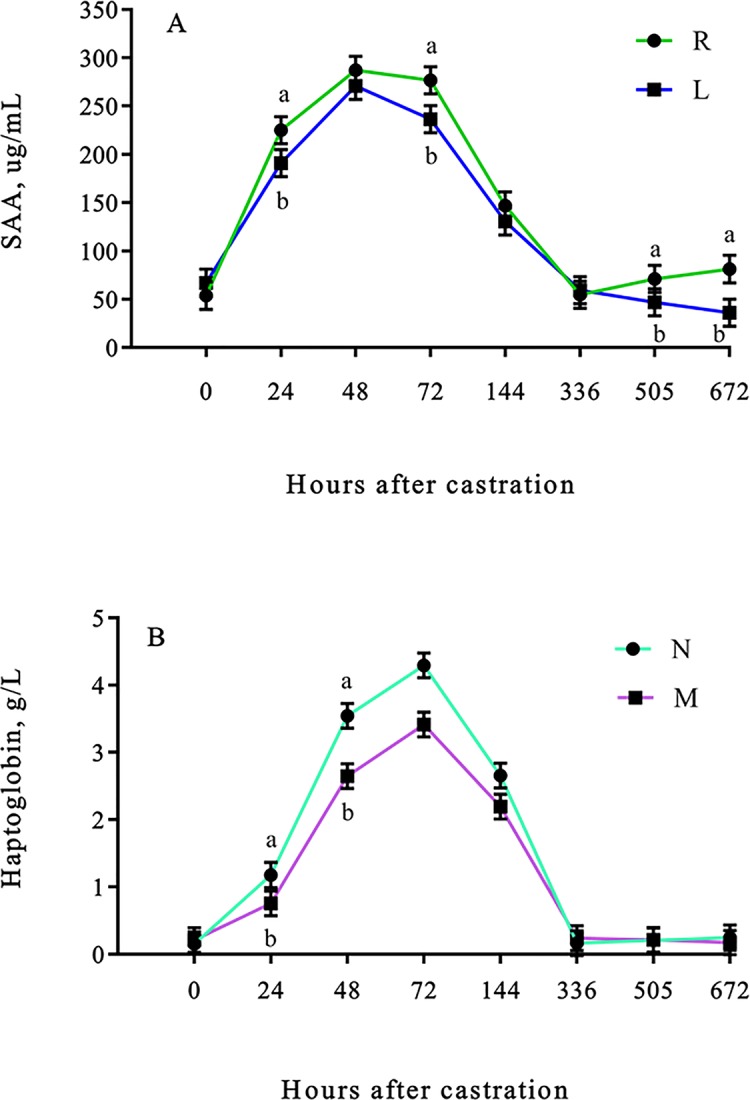
Least square means (±SEM) for serum amyloid-A and haptoglobin of weaned Angus crossbred calves. (A) Serum amyloid-A (μg/mL) concentrations of weaned Angus crossbred calves with (L) or without (R) a lidocaine ring block and (B) haptoglobin (g/L) concentrations of weaned Angus crossbred calves with (M) or without (N) a single s.c. meloxicam injection. ^a-b^Least square means with differing superscripts differ (*P* ≤ 0.05).

The SAA concentrations were greater (lidocaine × time effect; *P* < 0.01) in R calves than L calves 24, 72, 505 and 672 h after castration (Fig 2A), while haptoglobin concentrations were greater (meloxicam × time effect; *P* = 0.01) in N calves than M calves 24 and 48 h after castration (Fig 2B).

#### WBC count

**Fig 3:**
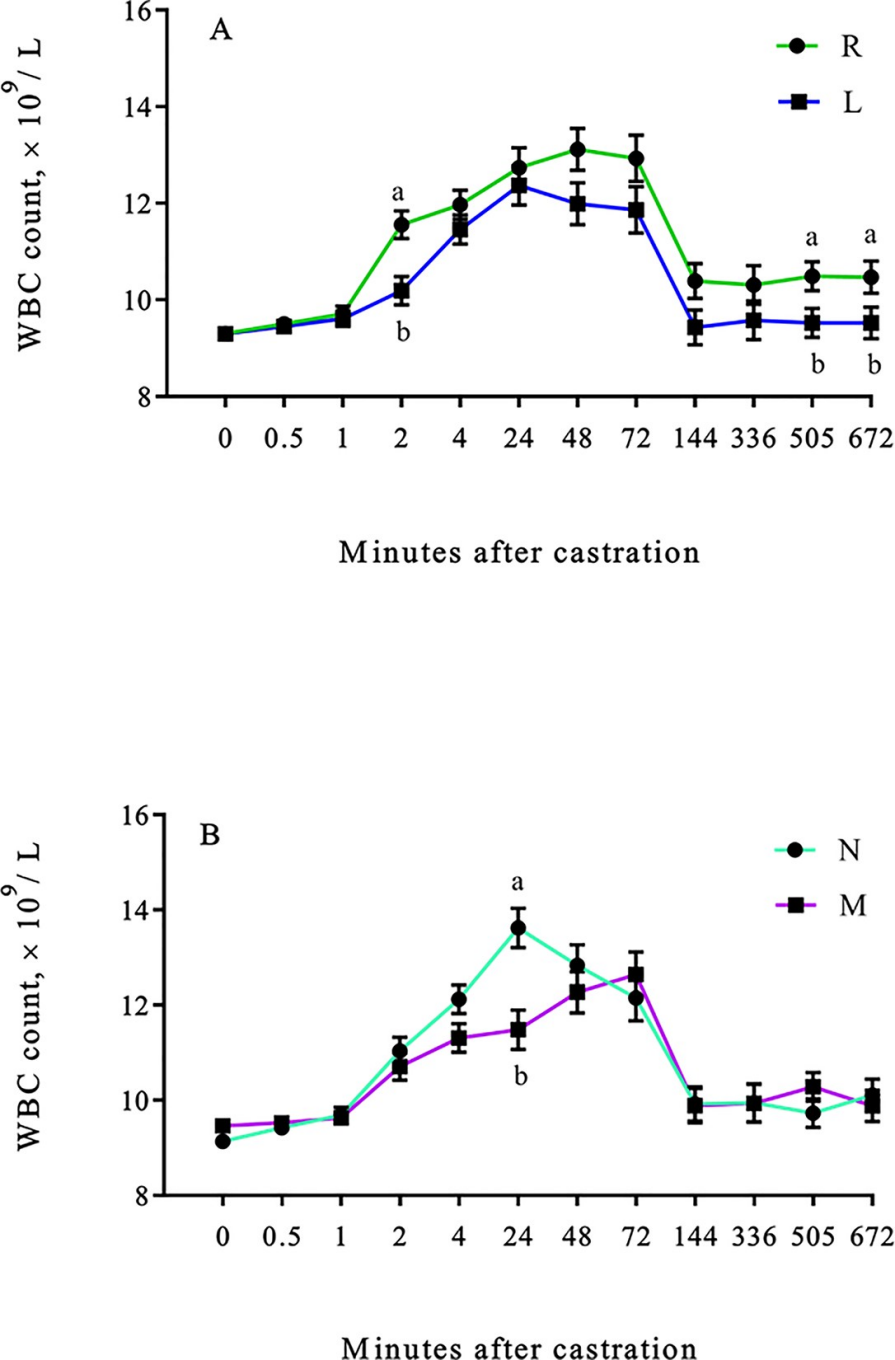
Least square means (±SEM) for WBC count of weaned Angus crossbred calves. WBC count (× 10^9^ /L) of calves (A) with (L) or without (R) a lidocaine ring block and (B) with (M) or without (N) a single s.c. meloxicam injection. ^a-b^Least square means with differing superscripts differ (P ≤ 0.05).

The WBC count was lower (lidocaine × time effect; *P* < 0.01) in L calves 2, 505 and 672 h after castration (Fig 3A), while the WBC count was lower (meloxicam × time effect; *P* < 0.01) in M calves 24 h after castration (Fig 3B).

#### Scrotal area temperature (SCT)

Scrotal temperature was greater (meloxicam × time effect; *P* = 0.01) in N (35.7 ± 0.02°C) calves than M (35.1 ± 0.02°C) calves 24 h after castration, while no differences (*P* ≥ 0.05) were observed at T0, 0.5, 1, 2, 4, 48, 72, 144, 336, 505 and 672 h after castration.

#### Scrotal circumference

**Fig 4:**
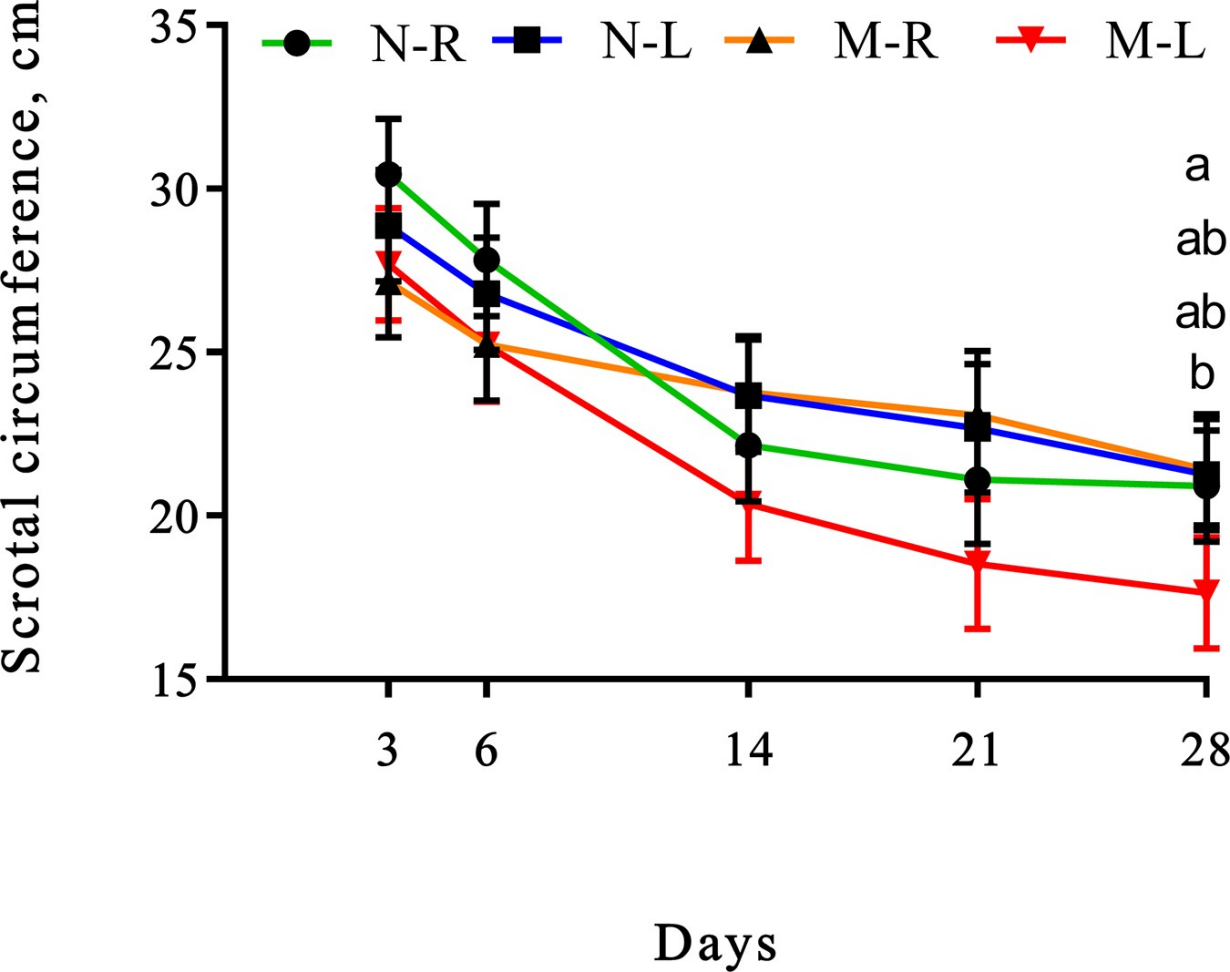
Least square means (±SEM) for scrotal circumference of weaned Angus crossbred calves. ^a-b^Least square means with differing superscripts differ (P ≤ 0.05).

Scrotal circumference had a triple interaction (lidocaine × meloxicam × time; *P* = 0.03), where M-R calves had greater scrotal circumference than M-L calves on d 28 (672 h) after castration (Fig 4).

Scrotal circumference of calves with (L) or without (R) a lidocaine ring block and with (M) or without (N) a single s.c. meloxicam injection. ^a-b^ Least square means with differing superscripts differ (P ≤ 0.05).

Lidocaine blocks sodium channels of nerve fibers which are necessary for the creation of action potentials, which carry nociceptive information to the dorsal horn. Lidocaine has an onset of action of 5 to 10 min after administration and a duration of action between 60 and 120 minutes. Epinephrine is a vasoconstrictor, commonly used as a local anaesthetic adjunct as it has the advantage of delaying the absorption and reducing the toxicity of local anaesthetics. Epinephrine counteracts the vasodilating effects of lidocaine, therefore increasing the duration of action of lidocaine, however it has the potential of causing localized ischemia [6].

The combination of lidocaine with epinephrine used in the present study was effective at reducing cortisol concentrations up to 90 min after administration (Fig 1A), which is equivalent to 60 min after castration, as the lidocaine ring block was administered 30 min prior to castration. Similar findings include a reduction in cortisol concentrations after an intra-testicular injection of lidocaine without epinephrine up to 75 min post castration when injected 20 min before surgical castration [44], and up to 0.25 to 1.5 hours after burdizzo and surgical castration when administered 15 min prior to castration [45] in 5.5 mo old dairy calves. In addition, a reduction in the peak cortisol response was reported after an intra-testicular and scrotal injection of lidocaine, without epinephrine, administered immediately before castration in 3 mo old dairy calves [11]. Contrary to our findings, intra-testicular lidocaine administered 15 min before castration, reduced the cortisol response for band and ring castration, but had little effect on surgical pull or surgical cut castration in 2 to 4 mo old dairy calves [46]; while lidocaine applied subcutaneously as a ring block 20 min before castration, had no effect on the cortisol response and was associated with a second increase in cortisol concentrations 120 min after surgical castration in 2 to 3 mo old dairy calves [12].

Differences between our study and studies that did not observe a lidocaine effect on cortisol concentrations [12,46] could be due to differences in the type of lidocaine used. However, previous studies have also reported a reduction in cortisol concentrations using lidocaine without epinephrine [10,11]. Moreover, differences observed between studies may be due to differences in the route of administration, as calves in the present study received lidocaine into each spermatic cord and around the neck of the scrotum, while calves in the previous studies only received an intra-testicular or a subcutaneous injection of lidocaine around the neck of the scrotum. In addition, calves in the present study were older, therefore the cortisol response is likely greater than in younger calves due to greater tissue damage at the time of castration [13]. Non-steroidal anti-inflammatory drugs (NSAIDs) inhibit COX enzymes which convert arachidonic acid from damaged cells into prostaglandins which are pro-inflammatory substances [8]. Previous studies have reported a reduction in cortisol concentrations 90 min after castration when flunixin meglumine was administered 20 min before surgical castration in comparison to un-medicated 2 to 3 mo old dairy calves [12], and a reduction in cortisol area under the curve when ketoprofen was administered 20 min before surgical castration in comparison to un-medicated in 5.5 mo old dairy calves [10]. In the present study, lidocaine reduced cortisol concentrations minutes after castration while meloxicam reduced cortisol concentrations days after castration (Fig 1B). However, we did not observe a lidocaine and meloxicam interaction for cortisol concentrations, which is contrary to previous studies and a review paper where the combination of an analgesic and an anaesthetics was more effective at reducing the cortisol response than either drug alone [3,11,46]. Lack of a lidocaine and meloxicam interaction could be due to the drugs acting at different time points as lidocaine had an effect prior to the effect observed for meloxicam.

Pro-inflammatory cytokines stimulate the production of acute phase proteins (APPs) in response to inflammation, infection, trauma or stress [47]. SAA has been previously reported to increase after inflammatory diseases [48], viral [49], and bacterial infections [50] in cattle, however few studies have assessed the SAA response in cattle after castration [35]. Lidocaine has been reported to inhibit pro-inflammatory cytokines, while stimulating the production of anti-inflammatory cytokines [51], which could explain the reduction in SAA and haptoglobin concentrations observed in calves receiving lidocaine. The ability of lidocaine to block nerve impulses is short, however, it seems that the anti-inflammatory effect lidocaine had on cytokines was sufficient to produce differences in SAA concentrations up to 21 (505 h) and 28 (672 h) d after castration.

Meloxicam had an effect on haptoglobin concentrations, but no effect was observed for SAA concentrations. Several studies have reported a reduction in the haptoglobin response after burdizzo and surgical castration in calves receiving an NSAID [10,15,44,52]. This is an interesting finding as we would expect lidocaine and meloxicam to have a similar effects on both SAA and haptoglobin. Similar findings were reported in a previous study where meloxicam was able to reduce the haptoglobin response but not the SAA response to castration and branding [53]. The author speculated that NSAIDs might not have the same effect on the response of different APPs, which was also observed in the current study for local anaesthetics. Differences observed in the haptoglobin and SAA response could be due to the ability of cytokines to activate two different acute phase protein genes within the liver through different binding proteins [54] or due to differences in the effect that analgesic and/or anaesthetic agents may have on the production of cytokines and glucocorticoids which have the ability of stimulating the APP response [55].

Meloxicam and lidocaine were able to reduce the WBC count after castration. This is in agreement with a previous study reporting a reduction in leukocytosis and neutrophilia in 3 mo old dairy calves receiving lidocaine and flunixin meglumine before surgical castration [11]. Although values in the present study were within the normal range (4–12 × 10^3^/μL) [42] for the majority of the sampling time points, values above this range were observed in R calves 48 h and in N calves 24 h after castration. Both lidocaine and meloxicam were able to reduce the leukocyte response, however differences were observed at different time points after castration, similar to the results observed for cortisol.

In the present study, scrotal circumference was measured as a proxy for inflammation. Scrotal size (measured using calipers), has been previously reported to increase the day of castration and to peak on d 2 and 3 after surgical castration in 25-day-old beef calves [56]. Greater scrotal circumference has also been previously reported in burdizzo castrated calves compared to control calves on d 7 after castration, while burdizzo castrated calves receiving lidocaine had greater scrotal circumference than control 5.5 mo old dairy calves on d 14, 21 and 35 after castration [10]. The author of the previous study suggested that the administration technique (intra-testicular) and the presence of lidocaine in the testicles could have caused greater inflammation. Contrary to the previous findings, in present study calves that received meloxicam in combination with lidocaine had lower scrotal circumference than calves that only received meloxicam, indicating that lidocaine was effective at reducing scrotal inflammation. However, caution should be taken when comparing studies as administration of lidocaine and castration techniques differ. This is the only parameter that presented a meloxicam and lidocaine interaction, and it is similar with the findings observed for WBC counts and SAA concentrations, where a lidocaine effect was observed on d 28. As indicated previously this could be associated with the ability of lidocaine to stimulate anti-inflammatory cytokines and to inhibit pro-inflammatory cytokines [51]. The mechanisms by which local anaesthetics act as anti-inflammatory agents have been attributed to the inhibitory effect that these drugs have at different stages of the inflammatory cascade [57], however to our knowledge there are no studies reporting a long-lasting local anaesthetic effect after a single administration.

#### Pain behaviours

**VAS.** The VAS scores of L (3.1 ± 0.09 cm) calves were lower (lidocaine effect; *P* < 0.01) than R (6.8 ± 0.09 cm) calves during castration (Table 2).

**Table 2:** Least square means (± SEM) of visual analog scale (VAS), leg movement, vocalizations and head movement during surgical castration and feeding behaviour after castration of weaned Angus crossbred calves with (M) or without (N) a single s.c. meloxicam injection and with (L) or without (R) a lidocaine ring block^1^.

	Treatment^2^							
	R		L				*P-Value*	
Item	N	M	N	M	*SEM*^3^	MEL	LID	MEL × LID
VAS, cm	6.6	2.8	7.0	3.5	0.12	0.26	<0.01	0.60
Leg movement, n	20.4	8.1	20.0	9.0	0.21	0.79	<0.01	0.50
Vocalization, n	0.21	0.0	0.33	0.21	0.06	0.39	0.34	0.69
Head movement, cm	2017^a^^b^	1604^b^	2346^a^	922^b^^c^	3.8	0.45	<0.01	0.07
Feeding behaviour								
Dry matter intake, kg/d	8.4	8.5	7.9	8.7	0.27	0.65	0.10	0.22
Feeding time, min/d	196	197	191	203	6.2	0.92	0.29	0.35
Feeding rate, g/min	44.8	45.4	44.1	45.3	1.21	0.75	0.47	0.79
Meal frequency, meal/d	10.7	11.4	11.3	11.5	0.37	0.33	0.09	0.56
Meal duration, min/meal	20.3	18.1	18.8	19.2	0.88	0.80	0.34	0.08
Meal size, kg/meal	0.86	0.79	0.79	0.83	0.04	0.62	0.80	0.14

^a-c^Least square means within a row with differing superscripts differ (*P* ≤ 0.05).

^1^Values in the table correspond to the means of visual analog scale (VAS), leg movement, vocalizations and head movement at the time of procedure; and feeding behaviour of week 1, 2, 3 and 4 after castration.

^2^Provision of anaesthesia administered 30 min prior to castration: R: no lidocaine; L: lidocaine; and provision of analgesia administered s.c. 30 min prior to castration N: no meloxicam; M: meloxicam.

^**3**^Values in the table correspond to nontransformed means; however, the SEM and the *P-*values correspond to ANOVA analysis using square root + 1 transformation for VAS, leg movement, vocalizations and head movement.

### Escape behaviours

#### Head movement

The head movement distance in L (1263 ± 2.1 cm) calves was lower (lidocaine effect; *P* < 0.01) than R (2181 ± 2.2 cm) calves during castration.

#### Leg movement

Leg movement frequencies in L (8.5 ± 0.15 n) calves were lower (lidocaine effect; *P* < 0.01) than R (20.2 ± 0.15 n) calves at the time of castration.

#### Accelerometers and head gate

**Fig 5:**
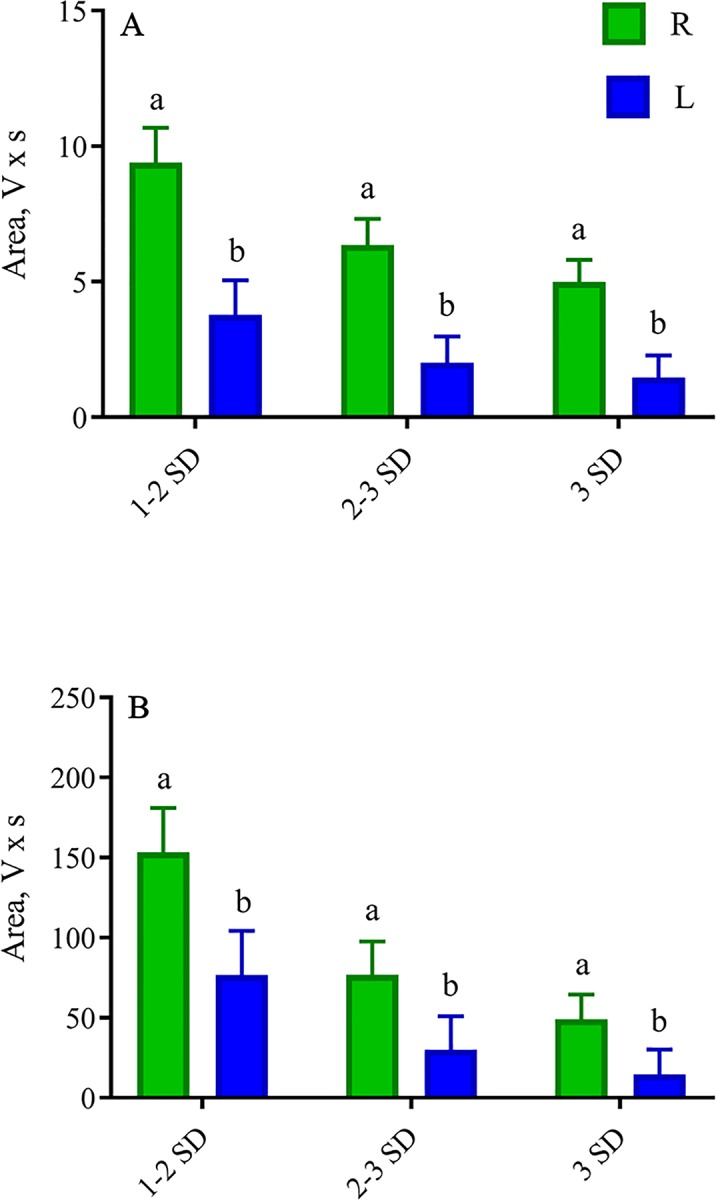
Least square means (±SEM) for total area of accelerometer and head gate data during castration. Total area (V × s) between ± 1 SD, ± 2 and ± 3 SD of (A) accelerometers and (B) head gate during surgical castration of weaned Angus crossbred calves with (L) or without (R) a lidocaine ring block injection. ^a-b^Least square means with differing superscripts differ (*P* ≤ 0.05).

Escape response for total area ± 1SD, ± 2 SD, and ± 3 SD was lower (lidocaine effect; *P* < 0.05) in L calves than R calves for accelerometer in the chute (Fig 5A) and head gate (Fig 5B) data. Number of accelerometer peaks 3 SD above and below the mean were lower (lidocaine effect; *P* < 0.05) in L (32 ± 1.0 n) calves than R (86 ± 1.0 n) calves, but no meloxicam or lidocaine effects (*P* > 0.10) were observed for accelerometer peaks between 1 and 2 SD and 2 and 3 SD above and below the mean No meloxicam or lidocaine effects (*P* > 0.05) were observed for accelerometer peaks between 1 and 2 SD, 2 and 3 SD and 3 SD above and below the mean.

### Feeding behaviour

No differences (*P* > 0.05) were observed for feeding behaviour.

### Stride length

No differences (*P* > 0.05) were observed for stride length at T0, 0.5, 1, 2, 4, 48, 72, 144, 336, 505, and 672 h after castration (Table 3).

**Table 3:** Least square means (± SEM) of stride length, standing and lying behaviour and behavioural observations of surgically castrated weaned Angus crossbred calves with (M) or without (N) a single s.c. meloxicam injection and with (L) or without (R) a lidocaine ring block^1^.

	Treatment^2^							
	R		L			*P-Value*		
Item	N	M	N	M	*SEM*^*3*^	MEL	LIDO	MEL × LID
Stride length, cm	46.8	47.7	46.2	46.2	0.92	0.25	0.58	0.63
Standing and lying beh.								
Standing, %	47.9	47.0	48.2	45.6	0.06	0.62	0.79	0.18
Lying, %	52.4	53.0	51.9	53.0	0.14	0.87	0.79	0.14
Standing duration, min	125.4	98.7	95.7	117.4	0.50	0.54	0.66	0.11
Lying duration, min	66.3	59.8	65.8	57.7	0.23	0.73	0.06	0.51
Behavioural obs.								
Standing, min	94.6	81.9	83.1	89.1	0.76	0.64	0.81	0.20
Lying, min	16.2	27.8	27.4	20.7	1.24	0.80	0.64	0.18
Eating, min	19.8	15.4	26.8	25.3	0.73	0.08	0.56	0.68
Tail flicks, n	630	309	654	210	4.4	0.97	0.07	0.88
Foot stamping, n	4.1	5.2	2.6	1.8	0.43	0.07	0.84	0.47
Head turning, n	5.4	4.7	4.8	5.8	0.22	0.76	0.90	0.35
Lesion licking, n	0.6	0.7	0.8	0.8	0.16	0.99	0.78	0.84

^1^Values in the table represent the mean of T0, 0.5, 1, 2, 4, 24, 48, 72, 144, 336, 505, 672 h after castration for stride length, d 0 to d 28 after castration (excluding d 6, 14, 21 and 28) for standing and lying behaviour and d 0, 1 and 2 after castration for behavioural observations assessed for a 2 h period.

^2^ Provision of anaesthesia administered 30 min prior to castration: R: no lidocaine; L: lidocaine; and provision of analgesia administered s.c. 30 min prior to castration N: no meloxicam; M: meloxicam.

^3^Values in the table correspond to non-transformed means; however, SEM and *P*-values correspond to ANOVA analysis using arcsine transformation for standing and lying percentage, and square root + 1 transformed data for stride length, standing and lying duration and behavioural observations.

### Standing/Lying

Standing duration was greater (lidocaine × time effect; *P* < 0.01) in L (465 ± 31.7 min; 162 ± 29.0 min) calves than R (293 ± 30.7 min; 90 ± 27.9 min) calves on d 1 and 5 and lower in L (185 ± 28.3 min; 156 ± 27.0 min) than R (276 ± 30.7 min; 294 ± 26.2 min) calves on d 3 and 7 after castration. Lying duration was greater (lidocaine × time effect; *P* < 0.01) in R (131 ± 6.9 min; 120 ± 6.8 min; 79 ± 6.8 min) calves than L (92 ± 7.0 min; 103 ± 7.0 min; 54 ± 6.9 min) calves on d 3, 4 and 7, but no differences (*P* > 0.05) were observed on d 9, 12 and 13 after castration.

### Pen behaviour

No differences (*P* > 0.05) were observed for tail flicking, foot stamping, head turning, lesion licking, walking, standing or lying ventral.

The VAS scale is highly criticized due to its subjectivity; however VAS scores in the present study were similar to the results obtained with the accelerometers and the head gate. The findings for behaviours assessed during castration are similar to previous results which reported a reduction in pain related behaviours during clamp and surgical castration in calves receiving lidocaine compared to un-medicated 2 to 4 mo old dairy calves [46]. Lack of a meloxicam effect at the time is in agreement with a previous study that reported a lack of VAS scores and movement in the chute differences at the time of band and surgical castration with or without an i.m. injection of ketoprofen administered 30 min before castration [36]. Lack of differences at the time of castration could be due to the route of administration, as s.c. meloxicam administered 30 min prior to castration may not be enough time for meloxicam to reach therapeutic levels to cause a central analgesic effect. However, the compendium for injectable meloxicam recommends the administration of meloxicam 10 to 20 min prior to abdominal surgery.

Previous studies have reported an increase in standing behaviour following surgical castration compared to prior to castration [58] and in surgically castrated calves compared to sham calves [12,24]. Therefore, we would expect un-medicated calves to have a greater standing percentage and/or duration than medicated calves. If greater standing behaviour would have been observed during the first few days after castration in the L calves, this could have been attributed to the lidocaine injection, as lidocaine can cause local tissue irritation [6]. However, there was no clear pattern for standing and lying behaviour in the present study for L and R calves.

Lack of significant differences in behaviours such as tail flicks and standing/lying duration are likely due to delayed behavioural scoring. Changes in behaviour between treatments were expected immediately after castration; however, it was not possible to assess behaviour during this period of time due to collection of physiological samples, which took place up to 5 hours after castration.

### Pharmacokinetics of meloxicam

**Fig 6:**
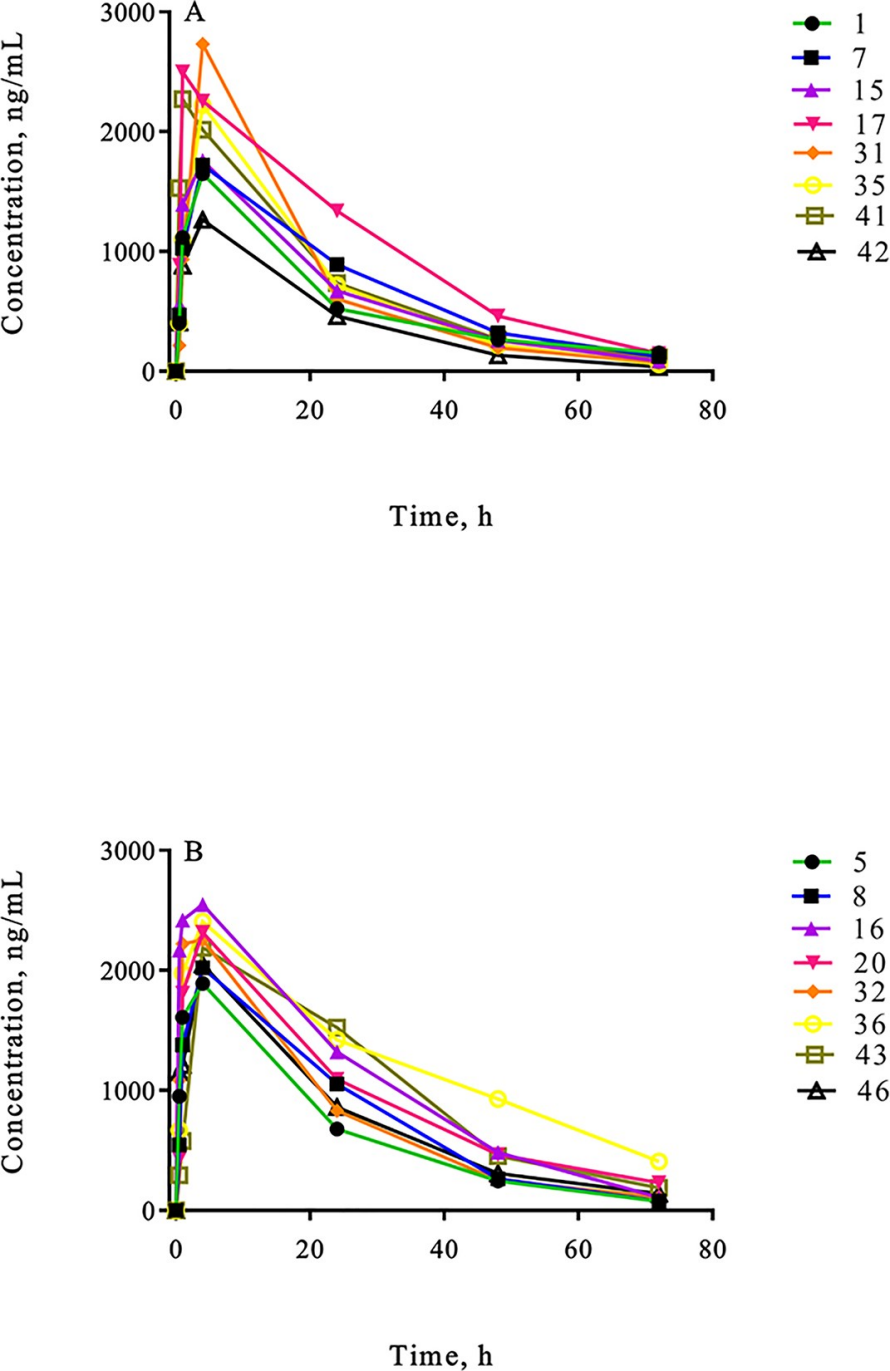
Plasma meloxicam concentrations of weaned Angus crossbred calves. Meloxicam concentrations in individual calves following a s.c. administration of (A) meloxicam and (B) meloxicam + lidocaine at the dose rate of 0.5mg/kg.

Measurable concentrations of meloxicam in plasma were obtained for s.c. administration of meloxicam or meloxicam + lidocaine (8 calves per treatment) throughout the 72 h monitoring period (Table 4, Fig 6).

**Table 4:** Pharmacokinetic (PK) parameters of meloxicam following s.c. administration of meloxicam at the dose rate of 0.5 mg/kg in calves (n = 8).

Item	M-R	M-L	*P*-Value
C_max,_ ng/ml	1959 ± 494	2200 ± 217	0.32
T_max_, h	2.83 ± 1.39	4.00 ± 0.00	0.15
*λ_z,_ 1/h	*0.041 ± 0.009	*0.040 ± 0.009	0.79
*λ_z_-HL	*16.10 ± 4.45	*16.65 ± 4.58	0.78
Vz_F, mL/kg	236.7 ± 84.4	181.0 ± 38.3	0.07
AUC_0-last,_ h × ng/mL	47768 ± 12203	^ψ^63797 ± 14290	0.03
AUC 0-∞, h × ng/mL	50286 ± 12607	^ψ^ 68082 ± 18473	0.03
AUC extrapolated, %	4.25 ± 3.35	5.17 ± 4.41	
AUMC_0-last,_ h^2^ × ng/mL	837909 ± 285975	^ψ^ 1250457 ± 483705	0.04
MRT0-∞, h	21.3 ± 4.70	24.10 ± 6.49	0.31
Cl_F, mL/h/kg	9.94 ± 2.76	^ψ^ 7.34 ± 1.79	0.03

PK parameters were determined using non-compartment modeling.

*Harmonic means and rest of the means are geometric (Geo mean) means ± SD.

^ψ^ Values are different between two treatments (M-R and M-L) at statistically significant level of *P* <0.05.

The area under the curve, which is an indication of total drug exposure, was significantly higher in the meloxicam + lidocaine group compared to the meloxicam group. In addition, meloxicam was excreted from the body (Cl_F) faster in the animals that received only meloxicam compared to calves that received meloxicam in combination with lidocaine. These were unexpected findings which show that lidocaine and/or epinephrine have an effect on meloxicam total drug exposure and clearance. We speculate that this could be due to a) the epinephrine in the lidocaine causing systemic vasoconstriction and therefore reduced rate of meloxicam elimination or that b) pain experienced at the time of castration in calves that did not receive lidocaine activated the autonomous nervous system, therefore increasing heart rate and blood pressure which could speed drug clearance [59]. However, the mechanisms by which lidocaine with epinephrine affects meloxicam are unknown.

The therapeutic concentrations of meloxicam have been reported to be 195 ng/ml in experimentally induced arthritis in the horse when using lameness score as an endpoint [60]. Meloxicam concentrations above 195 ng/ml were observed between 0 (immediately before castration) and 48 h after castration in the majority of animals in the present study. However, this study would require more frequent sampling time points to accurately determine the period of time meloxicam was above this particular concentration. Caution should be taken when extrapolating results obtained from horses, as there could be potential species differences in pharmacokinetics and pharmacodynamics [61]. Therefore, there is a need to identify therapeutic concentrations of meloxicam in cattle.

## Conclusions

Overall, lidocaine was effective at reducing physiological and behavioural indicators of pain (salivary cortisol, SAA, WBC, scrotal circumference, VAS, leg movement, head distance, escape response) while meloxicam only reduced physiological indicators of pain (salivary cortisol, haptoglobin, WBC, scrotal temperature and scrotal circumference). Parameters that weren’t affected by either drug included weight, hair cortisol, rectal temperature, feeding behaviour, stride length, standing and lying behaviour and pen behaviour. Meloxicam and lidocaine reduced the APP response, salivary cortisol concentrations and WBC counts at different time points after castration. Therefore, although we did not see a meloxicam and lidocaine interaction (with the exception of scrotal circumference), administering lidocaine in combination with meloxicam, may be more effective at mitigating pain associated with surgical castration, for a longer period of time. Further studies are needed to assess the therapeutic levels of meloxicam in cattle.

## Supporting information

S1 File Castration data. (XLSX)
